# Upregulation of Tumor Necrosis Factor-*α*-Induced Protein 8-Like 2 mRNA Is Negatively Correlated with Serum Concentrations of Tumor Necrosis Factor-*α* and Interleukin 6 in Type 2 Diabetes Mellitus

**DOI:** 10.1155/2017/4802319

**Published:** 2017-05-24

**Authors:** Yongliang Liu, Xinmei Wang, Yan Zhao, Peiqing Zhao, Lianqing Wang, Qiaoli Zhai, Xiaowei Zhang, Wenxiu Tian, Xinxin Xiang, Tao Li

**Affiliations:** ^1^Central of Translation Medicine, Zibo Central Hospital Affiliated to Shandong University, Zibo 255036, China; ^2^Department of Pathology, Zibo Central Hospital Affiliated to Shandong University, Zibo 255036, China; ^3^Department of Central Laboratory, Zibo Central Hospital Affiliated to Shandong University, Zibo 255036, China

## Abstract

**Background:**

Tumor necrosis factor-*α*-induced protein 8-like 2 (TIPE2 or TNFAIP8L2) is a negative regulator of natural and adaptive immunity. The role of TIPE2 in type 2 diabetes mellitus (T2DM) remains unknown, although TIPE2 plays key roles in preserving inflammatory homeostasis.

**Methods:**

TIPE2 expression was measured by Western blotting and real-time polymerase chain reaction (RT-PCR) in peripheral blood mononuclear cells (PBMCs) isolated from T2DM patients and healthy controls, and tumor necrosis factor-*α* (TNF-*α*), high-sensitivity C-reactive protein (hsCRP), interleukin 6 (IL-6), and other related biometabolic parameters were detected using a nephelometer or by ELISA. Differentiated THP-1 cells were exposed to siTIPE2 and TIPE2 adenovirus.

**Results:**

TIPE2 was significantly increased in PBMCs from T2DM patients compared with those from healthy controls and was negatively correlated with serum TNF-*α*, IL-6, and hsCRP concentrations but positively correlated with HbA1c and LDL-C in T2DM patients. High glucose treatment (50 mmol/L) can upregulate the expression of TIPE2 and cytokine secretion in differentiated THP-1 cells. siTIPE2 infection exacerbated the increased TNF-*α* and IL-6 concentrations in differentiated THP-1 cells under high glucose conditions (50 mmol/L), while infection with TIPE2 adenovirus reversed the increased TNF-*α* concentration.

**Conclusions:**

The present study indicates that TIPE2 may participate in T2DM by regulating TNF-*α* production.

## 1. Introduction

Type 2 diabetes mellitus (T2DM) is a chronic inflammatory metabolic disorder which is characterized by insulin resistance in the muscle and liver, *β*-cell failure, and hyperglycemia [1]. Intensive work has demonstrated an intimate correlation between insulin resistance and obesity [2, 3], and it is now frequently recognized that obesity-associated chronic inflammation prompts insulin resistance and *β*-cell dysfunction in diabetics [4, 5]. Insulin resistance interrupts the duties of insulin target organs, such as adipose tissue, which is crucial for lipogenesis, glucose metabolism, and adipokine secretion. Proinflammatory cytokines, including tumor necrosis factor *α* (TNF-*α*) and interleukin 6 (IL-6), may initiate c-Jun N-terminal kinase (JNK), which then limits Akt activity and hampers insulin signaling [6].

Inflammatory markers such as TNF-*α*, high-sensitivity C-reactive protein (hsCRP), IL-1*β*, and IL-6 are increased in the tissues and serum of T2DM patients [7–10]. TNF-*α* takes part in obesity-related systemic insulin resistance by suppressing insulin receptor tyrosine kinase activity in the adipose tissue and skeletal muscle [11]. Some evaluations discovered that human TNF-*α* prevents the insulin-dependent tyrosine phosphorylation of insulin receptor substrate 1 (IRS-1) and the insulin receptor in adipocytes and myeloid 32D cells [12]. Further, preparing 3T3-L1 adipocytes with TNF-*α* diminished glucose transporter 4 (GLUT4) expression and protein kinase B (Akt) activity [13]. Every one of these evaluations suggests that TNF-*α* is a key arbitrator of insulin resistance in obesity, and it could be a target in obesity-induced insulin resistance in individuals with T2DM. The tumor necrosis factor-*α*-induced protein 8 (TNFAIP8) family was distinguished not long ago; they are generated after TNF-*α* stimulation and NF-*κ*B actuation [7, 8], and they could be the molecular connection among TNF-*α*-mediated signaling and diabetes.

Tumor necrosis factor-*α*-induced protein 8-like 2 (TIPE2), an innovative gene belonging to the TNFAIP8 family, suppresses the liberation of proinflammatory cytokines, such as TNF-*α*, IL-4, IL-12, and IFN-*γ*, to maintain immune homeostasis [14]. TIPE2 is favorably produced in lymphoid tissues, and TIPE2-deficient mice suffer from chronic inflammatory diseases. In vitro experiments showed that TIPE2 inhibits NF-*κ*B and activating protein 1 activation and that TIPE2-deficient cells are hyper-reactive to Toll-like receptor (TLR) and T cell receptor (TCR) actuation [14–16]. Moreover, TIPE2 was found to be abnormally expressed in peripheral blood mononuclear cells (PBMCs) from patients with chronic hepatitis B or systemic lupus erythematosus (SLE) and from asthmatic children [17–21]. The data suggest that TIPE2 plays a vital role in the development of some certain chronic inflammatory diseases. However, it still remains unclear whether TIPE2 is involved in the development of T2DM. In the current evaluation, we looked at TIPE2 expression measurements and scrutinized the ties between *TIPE2* production and that of TNF-*α* and IL-6 to illuminate the precise roles of TIPE2 in T2DM.

## 2. Materials and Methods

### 2.1. Study Group

We enrolled 46 T2DM patients who attended Zibo Central Hospital (Shandong Province, China) and fulfilled the 1999 criteria of the World Health Organization for T2DM. The study excluded patients with type 1 diabetes, gestational diabetes, secondary diabetes, or severe liver and kidney disease. In addition, 30 age- and sex-matched blood donors who did not have any chronic or metabolic diseases were randomly recruited as a control group. All the patients signed an informed consent form prior to study participation. The characteristics of all the subjects are summarized in Table 1. The study was approved by the Ethics Committee of Zibo Central Hospital in accordance with the Declaration of Helsinki, and all procedures were approved by the Institutional Review Board of Zibo Central Hospital, Shandong University.

### 2.2. Cell Culture

THP-1 cells (ATCC, USA) were cultured in RPMI1640 medium containing 10% fetal bovine serum in a humidified atmosphere of 5% CO_2_ at 37°C. Macrophage-like cells were differentiated from parental THP-1 cells by induction with phorbol-12-myristate-13-acetate (PMA, 200 nmol/L; Sigma, USA) for 24 h and were then infected with TIPE2 adenovirus and siTIPE2 or treated with culture medium containing glucose (25, 35, and 50 mmol/L) for 24 h.

### 2.3. TIPE2 Short Interfering RNA (siRNA) and Adenovirus Construction

TIPE2-specific siRNA and a nonspecific negative control were purchased from Life Technologies (Life Technologies, USA). The TIPE2-expressing adenoviruses (Ad-TIPE2) were enlarged, titrated in 293 cells, and cleansed by cesium chloride techniques as detailed prior [22]. Transfection into THP-1 cells with siRNA or adenovirus was performed using a Lipofectamine®2000 Stealth/siRNA transfection according to the manufacturer's instructions (Life Technologies, USA). For adenovirus-mediated gene transfer, distinguished THP-1 cells were bared to adenoviral vectors at a multiplicity of infection of 100 for 24 h. Infection efficiency was generally higher than 60%, as judged by microscopic analysis of GFP expression. To detect the influence of TIPE2 on THP-1 cytokine products, TNF-*α* and IL-6 levels in cell culture supernatants were measured.

### 2.4. Detection of Biometabolic Parameters

The following biometabolic parameters were analyzed in T2DM patients: fasting glucose, HbA1c, total cholesterol, low-density lipoprotein cholesterol (LDL-C), high-density lipoprotein cholesterol (HDL-C), triglycerides, and hsCRP. The serum was separated and prepared according to standard protocols. The serum levels of fasting glucose, LDL-C, HDL-C, total cholesterol, and triglycerides were detected using standard commercially available, colorimetric-based enzymatic kits (Leadman, China). HbA1c was estimated using an HPLC method (Bio-Rad, USA). Concentrations of hsCRP were measured using a nephelometer (Model BN2, Japan).

### 2.5. Detection of the Serum Levels of TNF-*α* and IL-6 by ELISA

TNF-*α* and IL-6 concentrations in serum from T2DM patients or in culture supernatants of differentiated THP-1 cells were determined by ELISA (Dakewei Biotech, China) based on the manufacturer's directions. Serum samples were placed in a 96-well ELISA microplate that was prepared with captured antibody. The captured antibody-bound cytokines were determined with biotin-conjugated anti-TNF-*α* and anti-IL-6 antibodies and horseradish peroxidase- (HRP-) conjugated avidin. The reaction plates were read within 30 min at 450 nm using a microplate reader (Bio-Rad, China). TNF-*α* and IL-6 concentrations were determined based on a standard curve according to the manufacturer's instructions. Each sample was assayed in triplicate wells.

### 2.6. RNA Preparation from Peripheral Blood Mononuclear Cells

Peripheral blood from healthy controls was placed in sodium citrate-containing cell preparation tubes. PBMCs were isolated by gradient centrifugation over Lymphoprep (Axis-Shield, Scotland) and washed twice with phosphate-buffered saline (PBS). Total RNA was then extracted from PBMCs (5 × 10^6^) using Trizol (Invitrogen, USA), and the samples were treated with RNase-free DNase (Qiagen, Germany) to remove genomic DNA contamination according to the manufacturer's instructions.

### 2.7. Quantitative Real-Time Reverse Transcription Polymerase Chain Reaction

Total RNA (1 *μ*g) was reverse transcribed to cDNA with oligo (dT) primers with the RT system based on the manufacturer's directions. PCR was performed in a 25 *μ*l volume with 2.5 *μ*l of cDNA, 5 mM of MgCl_2_, 0.2 mM of dNTPs, 0.2 mM of each primer, 1.25 U of AmpliTaq DNA polymerase, and 1 *μ*l of 800 X SYBR Green I with the Mx3000 Multiple Quantitative PCR System (Stratagene, USA). RNA quality was ascertained by gel electrophoresis, and negative controls without RT were incorporated.

The following primers were used in this study: TIPE2 forward 5′-ACTGAGTAAGATGGCGGGTCG-3′ and reverse 5′-TTCTGGCGAAAGCGGGTAG-3′; 18S rRNA forward 5′-CGGCTACCACATCCAAGGAA-3′ and reverse 5′-GCTGGAATTACCGCGGCT-3′.

mRNA expression was measured with the comparative threshold cycle (Ct) technique. The Ct value of the housekeeping gene (18S rRNA) was removed from the Ct value of the target gene to gain the ΔCt. The normalized fold change in the target mRNA expression was shown as 2^−ΔΔCt^, where ΔΔCt = ΔCt_sample_ − ΔCt_control_. All of the real-time PCR were done in duplicate.

### 2.8. Western Blotting Analysis

The TIPE2 protein expression was detected by Western blotting. PBMCs and THP-1 were quickly harvested, rinsed thoroughly with PBS, and then homogenized on ice in lysis buffer. After centrifugation for 10 min at 4°C, the supernatant was used for Western blot analysis. Protein concentration was measured by a bicinchoninic acid protein assay kit (Beyotime, Shanghai, China). A total of about 60 *μ*g proteins from each sample was loaded onto SDS-PAGE gel. Proteins were transferred to nitrocellulose membranes. The membranes were incubated with 5% BSA in Tris-buffered saline containing Tween-20 for 1 h at room temperature, followed by incubation with anti-TIPE2 antibodies (Santa Cruz, CA, USA) overnight at 4°C. Specific reaction was detected using IRDye-conjugated second antibody for 1 h incubation and visualized using the Odyssey infrared imaging system (LI-COR Biosciences, Lincoln, NE, USA). Quantification of image density in pixel was performed by using the NIH ImageJ software (NIH, Bethesda, MD, USA).

### 2.9. Statistical Analysis

The significance of differences between groups was determined by a two-tailed nonparametric test. Pearson's correlation analysis was performed to determine the strength of the linear relationship between *TIPE2* mRNA expression level and the protein expression of TNF-*α*, IL-6, or other parameters in T2DM patients. All the statistical analyses were performed using GraphPad Prism 5.0 (GraphPad Software, USA). Two-tailed *P* < 0.05 was considered significant.

## 3. Results

### 3.1. TIPE2 Expression in PBMCs from T2DM Patients

**Figure 1:**
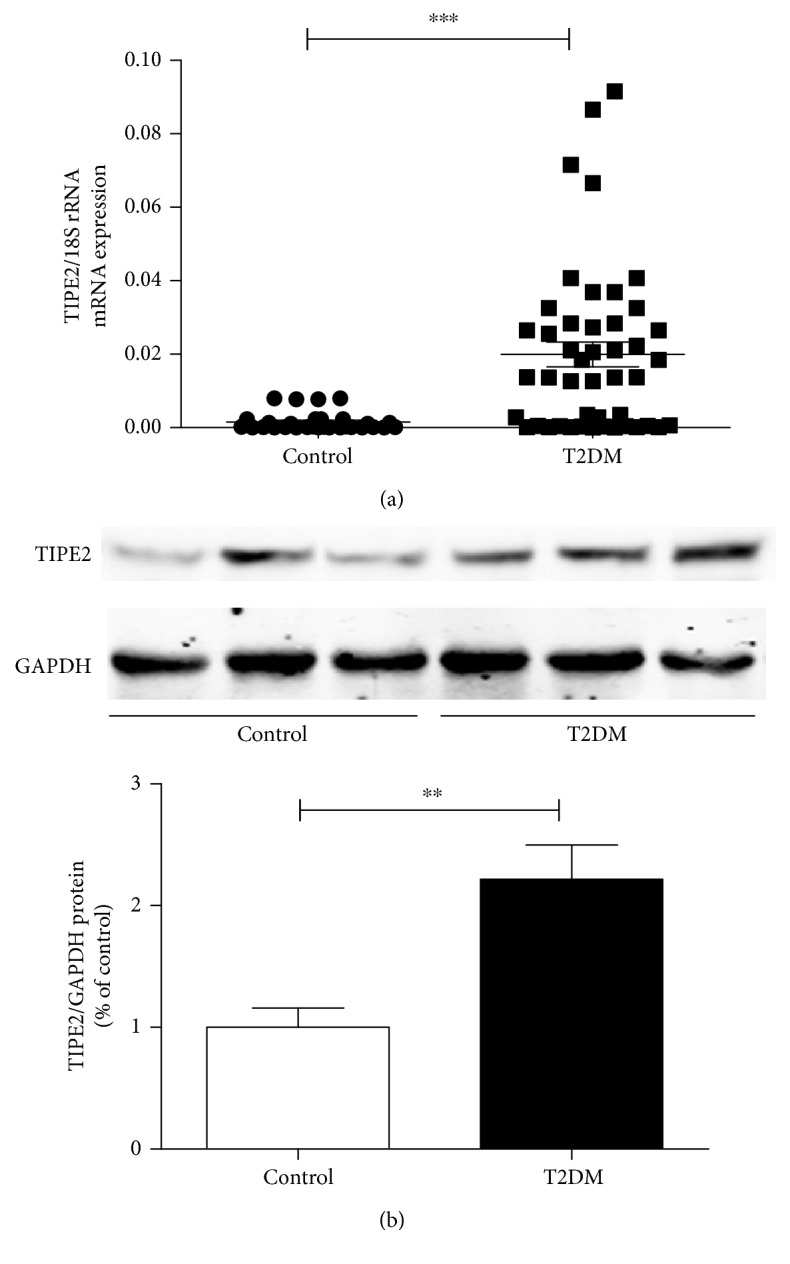
TIPE2 expression levels in PBMCs from individuals with T2DM and healthy participants. TIPE2 expression was determined by RT-PCR and Western blotting. The mRNA levels of *TIPE2* were obviously higher in PBMCs from T2DM patients (*n* = 46) compared with those from healthy controls (*n* = 30) (a). Every point depicts a specific participant, and the median value is indicated for each group. Statistical comparisons were performed using the Mann–Whitney *U* test. The TIPE2 protein levels were obviously upregulated in PBMCs from T2DM patients compared with those from healthy controls (b). Representative results of three individual experiments were shown for the TIPE2 expression in PBMCs. ^∗∗∗^*P* < 0.001 versus control; ^∗∗^*P* < 0.01 versus control.

Quantitative RT-PCR analysis discovered that *TIPE2* mRNA expression was higher in PBMCs from 46 T2DM patients compared to those from 30 healthy controls. There was a substantial escalation in the mean *TIPE2* mRNA expression in T2DM individuals in contrast to the controls (*P* < 0.01; Figure 1(a)). Consistent with the expression of TIPE2 mRNA level, the Western blotting also showed that the TIPE2 protein expression was increased in PBMCs from T2DM patients (*P* < 0.01; Figure 1(b)). These findings imply a possible role for *TIPE2* in the pathogenesis of T2DM.

### 3.2. Correlation between the mRNA Level of TIPE2 and the Serum Levels of TNF-*α*, IL-6, and hsCRP in T2DM Patients

**Figure 2:**
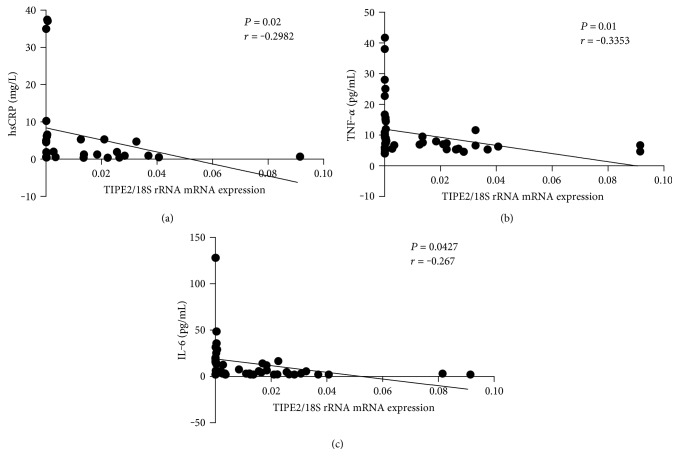
Correlation analysis of *TIPE2* mRNA expression and hsCRP, TNF-*α*, and IL-6 concentrations in participants with T2DM. *TIPE2* mRNA expression was determined by RT-PCR, whereas serum concentrations of hsCRP, TNF-*α*, and IL-6 were determined using a nephelometer or by ELISA. Pearson's correlation analysis was used for statistical comparisons. A negative correlation was found between *TIPE2* mRNA and hsCRP (a), TNF-*α* (b), and IL-6 (c) concentrations in T2DM patients. Every point shows information from a specific participant.

To further investigate the role of TIPE2 in diabetes, we studied the relationship between *TIPE2* mRNA expression and the serum concentrations of hsCRP, TNF-*α*, and IL-6. *TIPE2* mRNA expression in PBMCs from T2DM individuals was negatively linked with hsCRP (*P* = 0.02, *r* = −0.2982), suggesting that elevated *TIPE2* expression could be initiated by escalated levels of inflammatory elements (Figure 2(a)). Moreover, *TIPE2* mRNA expression was negatively correlated with TNF-*α* (*P* = 0.01, *r* = −0.3353) and IL-6 (*P* = 0.0427, *r* = −0.267), suggesting that *TIPE2* may inhibit TNF-*α* and IL-6 production (Figures 2(b) and 2(c)).

### 3.3. Effects of Glucose Levels on the Expression of TIPE2 and Cytokine Secretion by THP-1 Cells

**Figure 3:**
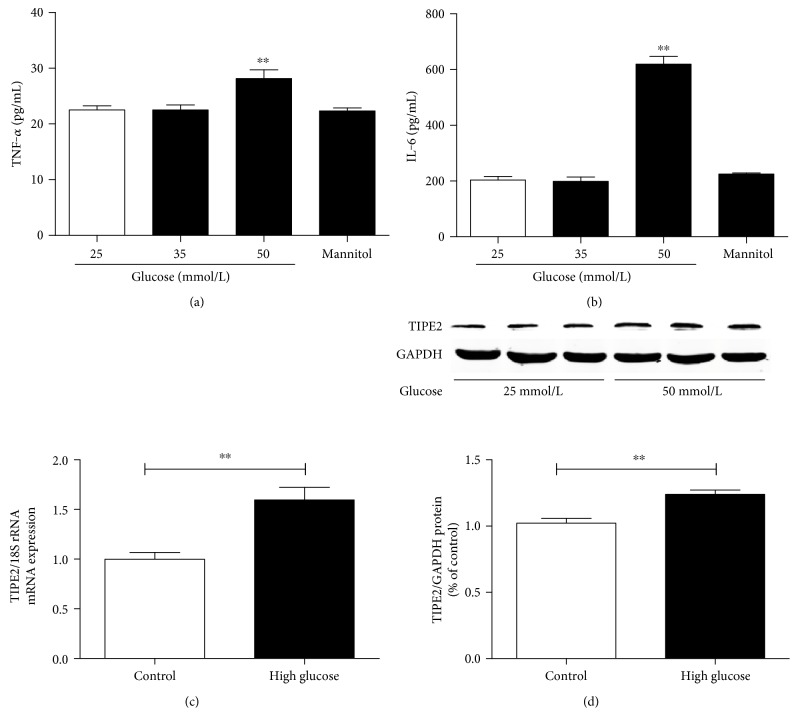
The effects of glucose levels on TIPE2 expression and TNF-*α* and IL-6 release from differentiated THP-1 cells. The concentrations TNF-*α* and IL-6 in the culture supernatant from differentiated THP-1 cells were determined by ELISA, whereas TIPE2 expression was determined by RT-PCR and Western blotting. TNF-*α* and IL-6 were significantly increased in culture supernatant from differentiated THP-1 cells exposed for 24 h to 50 mmol/L glucose, not 35 mmol/L glucose. The osmotic control mannitol had no effect on TNF-*α* or IL-6 levels (a, b). Compared to the normal glucose (25 mmol/L), high glucose (50 mmol/L) obviously increased the mRNA and protein expression levels of TIPE2 (c, d). Representative results of three individual experiments were shown for the TIPE2 expression in THP-1.^∗∗^*P* < 0.001 versus 25 mmol/L glucose (control) alone. The data are shown as mean ± SEM of 3 separate experiments.

TNF-*α* levels and IL-6 release were not affected in adherent differentiated THP-1 cells stimulated with high glucose (35 mmol/L) for 24 h. However, the concentrations of TNF-*α* and IL-6 were obviously increased in the culture supernatants of differentiated THP-1 cells exposed to 50 mmol/L glucose (Figures 3(a) and 3(b)). The osmotic control mannitol had no effect on TNF-*α* or IL-6 levels. Thus, we selected 50 mmol/L glucose as the treatment concentration for the following experiments.

As shown in Figures 3(c) and 3(d), high glucose (50 mmol/L) treatment also can increase the mRNA and protein expression levels of TIPE2 in differentiated THP-1 cells. These results show that high glucose can regulate TNF-*α* and IL-6 release and the expression of TIPE2 in vitro.

### 3.4. Effects of TIPE2 on Cytokine Secretion by THP-1 Cells

**Figure 4:**
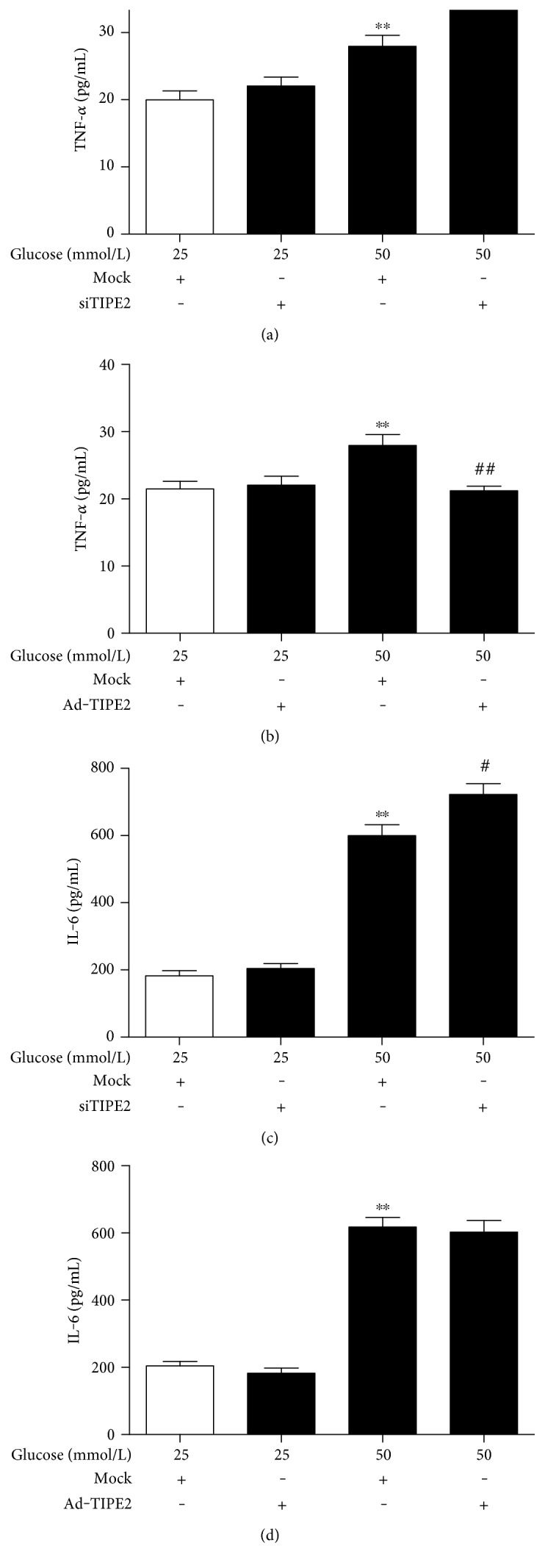
Effects of TIPE2 on cytokine secretion by THP-1 cells. The concentrations TNF-*α* and IL-6 in the culture supernatant from differentiated THP-1 cells were determined by ELISA. Under high glucose conditions (50 mmol/L), siTIPE2 infection exacerbated the increased TNF-*α* and IL-6 concentrations (a, c), while Ad-TIPE2 infection reversed the increased TNF-*α* concentration and had no obvious effect on the increased IL-6 release in differentiated THP-1 cells (b, d). ^∗∗^*P* < 0.001 versus 25 mmol/L glucose alone. ^#^*P* < 0.05 versus 50 mmol/L glucose + siTIPE2. ^##^*P* < 0.001 versus 50 mmol/L glucose + Ad-TIPE2. The data are shown as mean ± SEM of 3 separate experiments.

To further confirm the underlying relationship of TIPE2 and cytokine secretion, we designed the ectopic expression adenovirus TIPE2 (Ad-TIPE2) and TIPE2-specific siRNA (siTIPE2) to change the expression of TIPE2. Under high glucose conditions (50 mmol/L), siTIPE2 infection exacerbated the increased TNF-*α* and IL-6 concentrations in differentiated THP-1 cells (Figures 4(a) and 4(c)), and Ad-TIPE2 infection reversed the increased TNF-*α* concentration (Figure 4(b)), while Ad-TIPE2 infection had no obvious effect on the increased IL-6 release (Figure 4(d)). These results suggest that TIPE2 can regulate TNF-*α* and IL-6 release via a negative feedback mechanism.

### 3.5. Positive Correlation between the mRNA Level of TIPE2 and the Serum Levels of HbA1c and LDL-C in T2DM Patients

**Figure 5:**
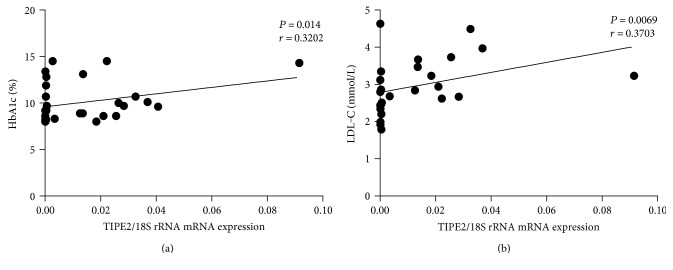
Correlation analysis between the mRNA levels of *TIPE2* and the serum concentrations of HbA1c and LDL-C in T2DM patients. *TIPE2* mRNA expression was determined by RT-PCR, and the serum concentrations of HbA1c and LDL-C in T2DM patients were determined by HPLC and standard colorimetric-based enzymatic kits, respectively. Pearson's correlation analysis was done. A positive correlation was found between *TIPE2* mRNA and HbA1c in T2DM patients (a). There was a tendency for a positive correlation between the mRNA levels of *TIPE2* and the serum concentrations of LDL-C in T2DM patients (b). Every point shows information from one participant.

We analyzed the association between *TIPE2* mRNA and metabolic parameters in T2DM patients. And then, a positive correlation was found between *TIPE2* mRNA expression and HbA1c (*P* = 0.014, *r* = 0.3202) and LDL-C (*P* = 0.0069, *r* = 0.3703) (Figures 5(a) and 5(b)), which are indicators of the status of T2DM. There were no substantial correlations among *TIPE2* mRNA expression and fasting glucose, total cholesterol, HDL-C, or triglycerides (information not displayed). These outcomes imply that TIPE2 could play a protective part in the occurrence of T2DM.

## 4. Discussion

TIPE2 is an innovative, necessary negative regulator of natural and adaptive immunity that is needed to preserve immune homeostasis and inhibit deleterious inflammatory reactions [14, 23]. TIPE2 was initially detected in inflamed mouse spinal cords, and TIPE2-deficient mice readily develop multiorgan inflammation, including in the lung. Sun et al. revealed that CD4^+^ and CD8^+^ T cell-mediated immune reactions were supplemented in the TIPE2^−/−^mice in contrast to the control mice, indicating the critical part of TIPE2 in T cell-mediated immunity [14].

The dysregulation of TIPE2 has been discovered to resolve heterogeneous human immunological and inflammatory diseases. It has been stated that *TIPE2* mRNA expression was substantially declined in individuals with SLE in contrast to healthy individuals and was negatively correlated with the SLE disease activity index (SLEDAI) and myxoma resistance protein 1 (MX1) mRNA expression levels in SLE participants [17]. Research by Feng Licon firmed that TIPE2 overexpression by adeno-associated virus prompted macrophage polarization to a M2 phenotype in vitro and in vivo in the SLE mouse model and substantially diminished SLE severity [9]. Xi et al. stated that *TIPE2* mRNA expression was substantially diminished in PBMCs from individuals with chronic hepatitis B in contrast to healthy people, and TIPE2 expression was negatively correlated with blood levels of ALT, AST, and total bilirubin (Tbil) and with HBV load in these patients [18]. A study by Li Kong showed decreased TIPE2 expression and enhanced TLR signaling in patients with HCV-mediated chronic hepatitis. Furthermore, Ma et al. reported that the lowered *TIPE2* mRNA expression in PBMCs from pediatric patients with asthma in contrast to healthy individuals was negatively linked to serum IL-4 and IgE levels and to eosinophil count [19].

However, Jia et al. showed that TIPE2 expression was highly upregulated in PBMCs from the chronic rejection group than in those from the healthy control group [21]. In addition, the mRNA and protein level of TIPE2 were significantly increased in renal biopsies of T2DM patients and in glomeruli from diabetic rats induced by streptozotocin (STZ) [20]. In accordance with these data, we observed that *TIPE2* mRNA expression was markedly increased in T2DM patients compared with controls, indicating that TIPE2 participates in T2DM.

In T2DM patients, some danger signals, such as systemic free fatty acid (FFA) flux, hyperglycemia, microhypoxia, ER stress, and higher mitochondrial ROS, can activate macrophages and other immune cells to make greater amounts of proinflammatory cytokines and various chemokines, which recruit additional macrophages [24, 25]. In turn, escalated levels of circulating proinflammatory cytokines and local inflammation in the pancreatic islets prompted insulin resistance. The pancreas also has a reserve of tissue-resident macrophages and immune cells; with increased glucose and FFA levels, these cells elicit inflammatory reactions that eventually lead to the apoptosis of *β*-cells and severely impair their insulin secretion [25–27]. As an innovative regulator of the immune response, TIPE2 may be induced by inflammation to modulate macrophage function and maintain immune homeostasis. The current evaluation revealed that *TIPE2* mRNA expression is negatively linked with the levels of hsCRP, an inflammatory factor, suggesting that increased TIPE2 expression may be caused by inflammatory elements in the early stage of T2DM, which would account for the increased *TIPE2* mRNA expression in T2DM patients in contrast to healthy individuals.

TIPE2, which belongs to the TNFAIP8 family, suppresses the liberation of proinflammatory cytokines, such as TNF-*α*, IL-4, IL-12, and IFN-*γ* [16]. Inflammatory markers, including IL-1*β* and TNF-*α*, are escalated in the serum and tissues of diabetics [8, 9], and TNF-*α* is a vital regulatory molecules of insulin resistance in T2DM; the neutralization of TNF-*α* might represent a strategy for combating obesity-induced insulin resistance in individuals with T2DM. In accordance with previous research, our study showed that the mRNA level of *TIPE2* was negatively linked with TNF-*α* and IL-6 in T2DM. In vitro experiments showed that siTIPE2 infection exacerbated the increased TNF-*α* and IL-6 concentrations, while Ad-TIPE2 infection reversed the increased TNF-*α* concentration in differentiated THP-1 cells under high glucose conditions (50 mmol/L). TIPE2 may be induced by the proinflammatory cytokines TNF-*α* and IL-6 and then inhibit the subsequent release of cytokines, especially TNF-*α*, in a negative feedback loop. We speculate that TIPE2 may participate in the pathogenesis of T2DM by modulating TNF-*α* production. Our study also identified a positive relationship between *TIPE2* mRNA expression and HbA1c and LDL-C levels. HbA1c is an indirect marker of dyslipidemia that points to long-term glycemic control; it has been connected to cardiovascular issues in T2DM patients [28–30]. Therefore, we suggest that TIPE2 is a crucial negative regulator of T2DM.

Thus, in the current evaluation, we contrasted *TIPE2* mRNA and protein expression levels in PBMCs from T2DM patients and healthy individuals and scrutinized the connections between *TIPE2* mRNA expression and the levels of inflammatory factors and metabolic parameters in vivo and in vitro. *TIPE2* mRNA and protein expression was upregulated in T2DM individuals and was negatively correlated with hsCRP, IL-6, and TNF-*α*, but positively correlated with HbA1c and LDL-C in T2DM patients. In vitro, high glucose treatment (50 mmol/L) can upregulate the expression of TIPE2 and cytokine secretion in differentiated THP-1 cells. siTIPE2 infection exacerbated the increased TNF-*α* and IL-6 concentrations, while Ad-TIPE2 infection reversed the increased TNF-*α* concentration in differentiated THP-1 cells under high glucose conditions (50 mmol/L). These outcomes imply that TIPE2 may take part in T2DM by regulating TNF-*α* production.

## Figures and Tables

**Table 1:** Characteristics of type 2 diabetes patients and control subjects.

	T2D patients (*n* = 46)	Control (*n* = 30)
Number of men/women	27/19	19/11
Age (years)	62.5 ± 14.4	60.3 ± 12.3
Race	Chinese	Chinese
Renal function (number of normal/abnormal)	30/16	—
Fasting glucose (mmol/L)	12 ± 3.6	—
HbA1c (%)	10.1 ± 2.1	—
AST (IU/L)	20.3 ± 8.9	—
ALT (IU/L)	20.0 ± 7.6	—
hsCRP (mg/L)	6.3 ± 10.8	—
Total cholesterol (mmol/L)	5.1 ± 1.1	—
LDL-C (mmol/L)	3.1 ± 0.83	—
HDL-C (mmol/L)	1.1 ± 0.31	—
Triglycerides (mmol/L)	1.72 ± 0.94	—

Unless indicated otherwise, values are given as the mean ± SD. AST: aspartate aminotransferase; ALT: alanine aminotransferase; hsCRP: high-sensitivity C-reactive protein; LDL-C: low-density lipoprotein cholesterol; HDL-C: high-density lipoprotein cholesterol.
